# Sealing ability of three different materials to repair furcation perforations using computerized fluid filtration method

**DOI:** 10.34172/joddd.2021.031

**Published:** 2021-08-25

**Authors:** Cemre Koç, Berna Aslan, Zuhal Ulusoy, Hasan Oruçoğlu

**Affiliations:** ^1^Department of Endodontics, Faculty of Dentistry, Başkent University, Ankara, Turkey; ^2^Bolu İzzet Baysal Oral Health Clinics, Bolu, Turkey; ^3^Private Practice, Sakarya, Turkey

**Keywords:** BC-RRM Putty, Endocem, Endosequence, Fluid Filtration, Furcation, Perforation, MTA

## Abstract

**Background.** The present study aimed to evaluate the sealing ability of three different calcium silicate-based materials in furcation perforations.

**Methods.** Seventy-six human mandibular molar teeth were selected. Perforations were created in the center of the pulp chamber floor. The experimental teeth were randomly divided into three groups (n=22). Perforations were repaired with MTA Angelus, Endocem MTA, or EndoSequence BioCeramic Root Repair Material Fast Set Putty (BC-RRM Putty). Microleakage of the different repair materials to be tested was measured by computerized fluid filtration method at 24- and 72-hour intervals.

**Results.** For each time interval, no statistically significant difference was observed between the groups. For Endocem MTA and BC-RRM Putty groups, the difference between the leakage values measured at both periods was not statistically significant (*P* > 0.05). However, there was a significant difference for the MTA Angelus group (*P* < 0.05).

**Conclusion.** All the calcium silicate-based materials used in the present study showed similar performance in repairing furcation perforations at 24- and 72-hour intervals.

## Introduction

A root perforation can be defined as a communication between the root canal system and supporting periodontal tissues of the tooth. The prognosis for teeth with root perforation usually depends on the time elapsed before treatment, size, location, and degree of periodontal damage. When root perforation occurs, it should be repaired as soon as it is made to prevent bacterial contamination, inflammation, and loss of attachment. Also, the location of root perforations is considered a detrimental factor in the treatment prognosis of the affected tooth. Perforation located in the coronal third of the root or the furcation area is more susceptible to apical migration of the gingival epithelium and periodontal pocket formation when compared to perforation in the middle or apical third of the root.^1-3^

Among various materials described over the years for perforation repair, MTA could be considered a material of choice due to its good biocompatibility and outstanding sealing ability. However, the use of MTA has some disadvantages, such as long setting time, discoloration potential, and difficult handling.^4^ To overcome these drawbacks, attempts have been made to develop new formulations of calcium silicate-based cements.

Recently, a new pozzolan-based MTA-derived material, namely Endocem MTA (Maruchi, Wonju-si, Korea), has been introduced. Endocem MTA has a short setting time and physical and biological properties comparable to MTA.^5^ Additionally, it results in less discoloration potential and higher washout resistance than the original form of MTA. Another newly developed premixed calcium silicate-based material is EndoSequence BioCeramic Root Repair Material Fast Set Putty (BC-RRM PuttyBrasseler, Savannah, GA, USA). This material has been shown to be biocompatible, with simple handling properties and a low risk of discoloration.^6^

Several studies evaluating leakage using various in vitro methods (dye penetration, bacterial, and fluid filtration) have shown that MTA is superior to other restorative materials for perforation repair.7-10 However, to the best of our knowledge, no studies have compared the sealing ability of new commercially available calcium silicate-based materials, Endocem MTA and BC-RRM Putty, with those of MTA Angelus (Londrina, Parana, Brazil) used as a reference material. The present study aimed to compare the sealing ability of Endocem MTA and BC-RRM Putty with that of MTA Angelus to repair furcation perforations using fluid filtration method at two different time intervals (24 and 72 hours). The first null hypothesis stated that there was no difference between two leakage values measured at 24 and 72 hours for each repair material. The second null hypothesis stated that there was no difference in leakage values measured for three different repair materials at both time intervals.

## Methods

### Tooth selection and preparation

Seventy-six three-rooted human mandibular left and right first molar teeth extracted for periodontal, prosthetic, or orthodontic reasons and without any restorations, cracks, or fractures were used. The collected teeth had a dentin thickness of 2.0‒2.5 mm at the furcation area to standardize the specimens. All the specimens were decoronated 3 mm above the cementoenamel junction and amputated 3 mm below the furcation area using a diamond saw under water cooling. Access cavities were prepared, and then root canal orifices were located. The canal orifices and the apical end of the roots were etched with 37% phosphoric acid gel (Scotchbond; 3M ESPE Dental Products, St Paul, MN) for 30 seconds, followed by the application of a single-bond adhesive system (Scotchbond; 3M ESPE Dental Products, St Paul, MN) and photopolymerization for 10 seconds. The root canal orifices and the apical end of each root were restored with a composite resin (Filtek Supreme XTE, 3M ESPE) and sealed with cyanoacrylate adhesive (Pattex; Henkel, Dusseldorf, Germany). Sixty-six teeth were randomly divided into three experimental groups (n = 22 for each group) according to the material used for perforation repair: Group 1: MTA Angelus; Group 2: Endocem MTA; Group 3: BC-RRM Putty. The remaining ten teeth served as positive and negative control groups. In the positive control group (n = 5), perforations were created and left unsealed. In the negative control group (n = 5), no perforation was created.

Perforations were created between the root canal orifices at the center of the pulp chamber floor using a #2 round high-speed bur under water cooling. Dentin residues in the perforation area were removed by rinsing with saline solution. The teeth were then placed in saline-soaked sponges in plastic cylinders to simulate clinical conditions. MTA Angelus and Endocem MTA were prepared by mixing the powder and liquid according to the manufacturer’s recommendations. BC-RRM Putty was used as received. The repair materials were then placed in furcation perforation areas using an MTA carrier and condensed with a plugger. A saline-moistened cotton pellet was placed in the pulp chamber. All the teeth were stored at 37°C and 100% humidity for 24 hours to allow the materials to set.

### Fluid filtration method

Microleakage of the different repair materials to be tested was measured by electronically monitoring the movement of air bubbles in the micropipette (Microcaps, Fisher Scientific, Philadelphia, PA, USA) by connecting to a computerized fluid filtration measurement device modified by Oruçoğlu et al.^11^ Acrylic blocks were first prepared to connect the samples to be tested to the system in the computerized liquid filtration method. Then,18-gauge stainless steel tubes were placed in the acrylic blocks, and the specimens were fixed to the acrylic blocks with a cyanoacrylate adhesive. The 18-gauge stainless steel tube was connected to a 25-μL glass micropipette through a polyethylene tube (Fisher Scientific, Pittsburgh, PA). The pressure regulator in the device was adjusted to approximately 2.0 atm (1.9738 atm). The micro-syringe in the computerized liquid filtration device formed air bubbles, and microleakage values (µL.cmH_2_O-^1^cm-^2^.min-^1^) of each specimen were obtained by automatically measuring the amount of air bubble movement in the glass micropipette. Microleakage values of all the specimens were measured at 24- and 72-hour intervals.

### Statistical analysis

Shapiro-Wilk test was used to determine whether the variables were suitable for normal distribution. Microleakage values were compared with nonparametric Kruskal-Wallis and Wilcoxon tests. The level of significance was set at *P* < 0.05.

## Results

In the positive control group, air bubble movement was too rapid to be measured due to the high fluid transport, whereas no fluid transport was recorded in the negative control group.

Mean leakage values of MTA Angelus, Endocem MTA, and BC-RRM Putty at 24 hours were 2.5721, 2.1356, and 1.6463, respectively. Mean leakage values of MTA Angelus, Endocem MTA, and BC-RRM Putty at 72 hours were 1.8112, 1.9955, and 1.4766. Although the highest leakage value was recorded in the MTA Angelus group at 24 hours, no statistically significant difference was observed between the groups (*P* = 0.052, *P* > 0.05). Although the highest leakage value was recorded in the Endocem MTA group at 72 hours, no statistically significant difference was observed between the groups (*P* = 0.519, *P* > 0.05) (Table 1).

**Table 1:** The analysis of the microleakage values of the groups in terms of the materials and time intervals (µL.cmH_2_O-^1^cm-^2^.min-^1^)

**Perforation repair material**	**Mean**		**Median**		**Standard deviation**		**P value**
	**24 h**	**72 h**	**24 h**	**72 h**	**24 h**	**72 h**	
MTA Angelus	2.5721	1.8112	2.4165	1.6875	±1.71	±1.50	0.001*
Endocem MTA	2.1356	1.9955	2.00	2.1250	±0.83	±1.02	0.828
BC-RRM Putty	1.6463	1.4766	1.1430	0.8750	±1.59	±1.59	0.506
P value	0.052	0.529					
*P < 0.05; Wilcoxon test.						

When the leakage values of the three repair materials at 24 and 72 hours were compared within the groups, leakage values measured at 72 hours were lower than those at 24 hours. For Endocem MTA and BC-RRM Putty groups, the difference between the leakage values measured at both time intervals was not significant (*P* = 0.828 and *P* = 0.506, respectively; *P* > 0.05). However, there was a significant difference in the MTA Angelus group (*P* = 0.001; *P* < 0.05) (Table 1).

## Discussion

The present study evaluated the sealing ability of Endocem MTA, BC-RRM Putty, and MTA Angelus at two different time intervals when used as furcation perforation repair materials. For all the materials, lower leakage values were obtained at 24-hour measurement than values measured at 72-hour. Only in the MTA Angelus group, significantly low values were found between the two time intervals. Thus, the first null hypothesis tested was partially accepted. All the three materials exhibited a similar ability to seal furcation perforations at 24 and 72 hours. Therefore, the second null hypothesis tested was accepted.

Various methods such as dye, bacteria, protein, radioisotope, and glucose penetration, and fluid filtration have been used in vitro to evaluate the leakage properties of materials.^12^ Among the mentioned methods, nondestructive and quantitative testing methods, the fluid filtration method has been reported to be more reliable than other methods.^13^ In the dye penetration method, it is not possible to measure the amount of leakage precisely and at different time intervals.^14^ In case of bacterial leakage in two-chamber models, potential routes of bacteria and antimicrobial properties of tested material could affect the results.^15^ The glucose leakage method is not particularly suitable for evaluating the sealing ability of MTA, Portland cement, and calcium hydroxide, which could directly react with glucose as a tracer.^16^ Liquid filtration method was first described by Derkson et al^17^ in 1986 to evaluate the sealing ability of temporary filling materials. Then, some modifications were made to the method in the field of endodontics.^13,18^ In the liquid filtration method, the sealing ability of root canal fillings can be evaluated from both apical and coronal aspects, and measurement of the same samples can be repeated at different time intervals because the samples are not destroyed.^19,20^ Therefore, the liquid filtration method was used to evaluate the sealing properties of different materials in the present study. Additionally, a pressure of 2 atm was applied to mimic physiological conditions.^21^ Pommel and Camps’s^22^ study showed that the measurement time should be as long as possible to obtain accurate measurement data. Wu and colleagues’^20^ study used the data at 3 hours due to uncertain correlation of measurements with clinical conditions. In vitro studies evaluating the sealing properties of the materials were not determined for an optimal period, and the application techniques of the materials or related materials were compared by themselves.^8,23-25^

In the present study, the sealing ability of the newly developed MTA derivative materials was evaluated at 24 and 72 hours. There was no significant difference between the leakage values measured at both time intervals. Although all the tested materials have different features, such as adding pozzolan particles (Endocem MTA), consisting of fine particles (BC-RRM Putty), and not containing calcium sulfate (MTA Angelus), they maintain a chemical composition similar to that of commercially available MTA. Thus, they may exhibit similar sealing ability when used as a perforation repair material. Similar to the present study results, another study evaluated the sealing ability and marginal adaptation of calcium silicate-based cements.^26^ The authors reported no significant difference in microleakage between the three different calcium silicate-based materials due to the similar compositions of the materials.

It has been suggested that hydroxyapatite crystals formed on the MTA surface fill the microscopic gaps between the MTA and dentin walls, and these hydroxyapatite crystals chemically bond to the collagen in the dentin.^27^ Reyes-Carmona and colleagues’^28^ study showed that the chemical bonding of MTA to dentin positively affected the sealing property of the material due to the formation of an apatite layer on the surface. However, the setting reaction and crystallization phase of the tested materials might not occur at the same time for each MTA derivative. Thus, leakage values measured at 72 hours were lower than those measured at 24 hours for all the materials; however, the difference was significant only in the MTA Angelus group in the present study. Additionally, the lower leakage values of all three materials at 72 hours compared to 24 hours can be attributed to the chemical bonding of the hydroxyapatite layer formed on the MTA surface with the collagen fibers in the dentin over time.

## Conclusion

Based on the results of the present study, there were no significant differences between the leakage values of all materials when used as furcation perforation repair materials. They showed similar behavior in preventing microleakage at both time intervals (24 hours and 72 hours). Only MTA Angelus did show significantly better sealing ability at 72 hours compared to 24 hours, which might be related to possible differences in setting reactions and crystallization phases of the tested materials.

## Authors’ Contributions

CK contributed to the study design, data acquisition, analysis, and interpretation, and writing the manuscript. BA participated in study design and writing the manuscript. ZU and HO participated in the acquisition of data. All authors read and approved the final manuscript.

## Acknowledgments

The authors thank Maruchi for providing the Endocem MTA in this study.

## Funding

The authors received no funding for this research.

## Competing Interests

The authors have no competing interests to declare.

## Ethics Approval

The present study was approved by the Ethics Committee of Faculty of Dentistry, Ankara University (No: 36290600/68).
